# Impact of non-farmacological methods on improvement cognitive function in epilepsy

**DOI:** 10.1192/j.eurpsy.2021.1107

**Published:** 2021-08-13

**Authors:** I. Blazhina, V. Korostiy

**Affiliations:** 1 Department Of Nervous Diseases Psychiatry And Medical Psychology, Bucovinian State Medical University, Chernivtsi, Ukraine; 2 Psychiatry - Narcology And Medical Psychology, Kharkiv National Medical University, Kharkiv, Ukraine

**Keywords:** Epilepsy, Cognitive disorders

## Abstract

**Introduction:**

The quality of life of patients with epilepsy, their social activity and functioning depends not only on the presence of epileptic seizures, but also on the level of cognitive decline.

**Objectives:**

The object of our study is impact of non-pharmacological methods on cognitive functions, decreasing of which deteriorates social activity in patients with epilepsy.

**Methods:**

We have studied the features of clinical and psychopathological manifestations in patients suffering from epilepsy. The study covered 27 patients who were in inpatient care. The following psychodiagnostic techniques were used: the Toronto Cognitive Assessment TorCA, the MOCA and the MiniMult tests, Patient Social Functioning Questionnaire. Currently, a group of patients with cognitive decline cause by epilepsy is undergoing remote cognitive training on one of an online platforms, under our observation. Correction occurs throughout regular daily performance, assessment of cognitive functions is carried out on three indicators: attention, memory and thinking

**Results:**

The following results of the study were observed: initially decreased memory in 88,8 % patients and the level of cognitive decline were directly proportional to the duration of the illness, this category of patients has reduced activity and limited social contacts.The result of the use of cognitive training is an increase in all three indicators.

**Conclusions:**

The results of the study indicate the need for further study of the features of cognitive disorders in epilepsy and the use of methods of psychotherapeutic correction.

